# Colonoscopy outcomes of primary screening negative participants highlight the missed diagnosis problem of colorectal cancer screening: an observational study from Yuexiu district in Guangzhou, China

**DOI:** 10.3389/fonc.2025.1642326

**Published:** 2025-10-21

**Authors:** Yu Liu, Yujing Fang, Yahui Xu, Shuang Wang, Yanping Wu, Kunhao Bai, Paul W. Bible, Qingjian Ou, Meixian Ye, Jiali Chen, Meiying Lu, Zhizhong Pan, Zhongjin Yao, Chenghua Gong, Desen Wan, Zhenhai Lu

**Affiliations:** ^1^ School of Public Health and Management, Guangzhou University of Chinese Medicine, Guangzhou, China; ^2^ Sun Yat-sen University Cancer Center, State Key Laboratory of Oncology in South China, Collaborative Innovation Center for Cancer Medicine, Guangzhou, China; ^3^ School of Public Health, Li Ka Shing Faculty of Medicine, University of Hong Kong, Hong Kong, Hong Kong SAR, China; ^4^ School of Public Health, Imperial College London, London, United Kingdom; ^5^ Department of Chronic Disease Prevention and Control, Yuexiu District Center for Disease Control and Prevention, Guangzhou, China; ^6^ Department of Computer Science, DePauw University, Greencastle, IN, United States

**Keywords:** colorectal cancer, screening, missed diagnosis, fecal immunochemical testing, high-risk factor questionnaire

## Abstract

**Objective:**

False negatives in colorectal cancer (CRC) screening remained a widespread concern, particularly given the notable incidence of false negative results from fecal immunochemical test (FIT). We aimed to investigate the missed diagnoses resulting from primary screening conducted in China that combined the high risk factor questionnaire (HRFQ) with double FITs.

**Methods:**

A retrospective cohort study was conducted in Yuexiu district of Guangzhou. Among 69,809 eligible participants who completed the primary screening between 2015 and 2021, we focused on the analysis of 527 subjects who had negative primary screening but underwent colonoscopy.

**Results:**

These individuals showed statistically comparable prevalence of overall colorectal neoplasms (CRN), advanced colorectal neoplasms (ACRN), and CRC in comparison with those having positive primary screening results (all *P*>0.05). When compared with subjects having negative primary screening results but no colonoscopy, screening negative participants with colonoscopy were more likely to be younger, possess higher education levels, and have one risk factor for CRC. A logistic regression analysis demonstrated that the missed diagnoses might attribute to the limited risk predictive ability of HRFQ for non-advanced adenoma (OR[95% CI]: 1.11 [0.98, 1.26]; *P* = 0.103), advanced adenoma (AA) (0.44 [0.38, 0.50]; *P* < 0.001), CRC (0.39 [0.29, 0.53]; *P* < 0.001), CRN (0.66 [0.59, 0.73]; *P* < 0.001) and ACRN (0.41 [0.36, 0.47]; *P* < 0.001).

**Conclusions:**

Subjects with negative primary screening results but having active screening willingness should consider an earlier colonoscopy due to HRFQ’s limited risk predictive ability for colorectal lesions, highlighting an urgency in re-assessment and improvement of the CRC risk scoring system.

## 1 Introduction

Colorectal cancer (CRC) remains a major challenge to global public health (1, 2). In China, the incidence and burden of CRC have been experiencing a rapid increase, with a trend of younger onset (3–5). In response to this growing threat, mass screening efforts, combining a high risk factor questionnaire (HRFQ) and double fecal immunochemical tests (FITs) as a primary screening strategy and colonoscopy as a diagnosis confirmation, have been implemented since 2006 to detect more early CRC cases or its precursor lesions (6). However, false negatives in CRC screening remain a significant concern, as they provide false reassurance to patients, leading to delayed diagnosis and more adverse prognosis (7, 8).

FIT is currently the most common primary method for CRC screening (8). However, it is also suffering from false negative results, owing to several inherent limitations, including its relatively low sensitivity in advanced adenoma (9), the controversy in cut-off value (8, 10, 11), and a low follow-up rate of colonoscopy, on the results (12). Multiple risk scoring systems like HRFQ have been introduced to increase the screening sensitivity and improve the missed diagnosis in high-risk persons with false negative FIT results (13, 14). However, even with the combined use of these screening methods, there still exists a great number of positive cases found from the non-high-risk population, revealing the inherent flaws in the current system (15, 16).

Further efforts in reducing missed diagnosis in CRC population screening should focus on the identification of participant-related risk factors contributing to false-negative outcomes in questionnaire assessment or FIT. These risk factors consist of age, sex, smoking, family history of CRC, BMI, and all kinds of medication et al. (17–19) The current HRFQ risk stratification does not include such variables and thus limits the discriminatory ability of colorectal neoplasms (20). Additionally, awareness of these factors can alert clinicians to the potential for false negatives, but such insights are insufficient to mitigate the issue without further innovations in screening strategies.

Shifting the focus from the well-studied, screen positive population, our study concentrated on a critical yet neglected subgroup that have negative primary screening results, to quantify their prevalence of colorectal neoplasms, to identify their features different from other risk stratification, and to explore the underlying reasons for the missed diagnosis at a unique prospective.

## 2 Materials and methods

### 2.1 Study design and population

The CRC screening in community allied third-grade class-A hospital (21) has been launched in Guangzhou of China since 2015 (22). The screening protocol (see below) was conducted in Guangzhou primary care units and their allied hospitals qualified for colonoscopy. Short message service (SMS) reminders of free CRC screening were sent to all permanent residents aged 40–74 years old (22). The primary care units were required to sensitize eligible people within their jurisdictions to first complete the HRFQ then undergo FIT twice. Participants with a positive result in any of the above mentioned tests were referred to undergo colonoscopy at designated allied hospitals through advice notes issued by the screening physicians. Additionally, participants with negative HRFQ and negative FIT results were also allowed for colonoscopy after their physicians’ approval. The primary care units were responsible for the follow-up of all these participants and a specialized online CRC screening system was established for data collection and management (23).

For the present study, data collected between 2015 and 2021 from Yuexiu District of Guangzhou (**Supplementary Figure S1**) were included. We focused on participants having colonoscopy outcomes but with negative results for both the HRFQ and FIT screens. All colonoscopy examinations were performed in the allied medical institutions including Sun Yat-sen University Cancer Center (SYSUCC), the largest oncology base in southern China.

### 2.2 Screening protocol

The screening strategy, maintained in this 7-year study, has been documented in previous studies, including ours (22–24). In brief, after providing a written informed consent, all participants were asked to complete the primary screening, that is, a HRFQ questionnaire followed by double FIT tests within two consecutive weeks. The HRFQ collected basic information of the participants (e.g., sex and age) and their medical history and health-related behavior associated with CRC cancer (e.g., alcohol drinking and smoking history). Participants were determined to be HRFQ positive if they had 1) personal history of cancer, 2) personal history of intestinal polyps, 3) first-degree relative(s) with CRC, or 4) two or more of the following risk factors: 4a) chronic diarrhea (diarrhea lasting for more than 3 months in the past two years, and each episode lasting for more than 1 week), 4b) chronic constipation (over the past two years, constipation lasting for more than 2 months), 4c) mucoid or bloody feces (over the past two years), 4d) history of chronic appendicitis or appendectomy, 4e) history of chronic cholecystitis or cholecystectomy, and/or 4f) major trauma or painful event in the past 20 years. In addition, a second test of FIT was required for every participant regardless of the result of the first. And a FIT test was considered positive when the hemoglobin concentration in the sample was ≥ 100 ng/ml, which corresponds to ≥ 20 μ g Hb/g feces. Eventually, a primary screening was defined as positive if any of the HRFQ or FIT test was positive. During the colonoscopy, polyps with a diameter < 5 mm were resected, if possible; any neoplasm with a diameter ≥ 5 mm was biopsied first and proceeded with polypectomy or colectomy depending on pathological report and feasibility of endoscopic surgery (23, 24). Abnormal findings in colonoscopy included benign lesions (i.e., intestinal polyp, enterocolitis, and non-adenomatous lesions), non-advanced adenoma, advanced adenoma, and CRC. Non-advanced adenoma was defined as adenomas with the following features: size < 10 mm, tubular histology and low-grade dysplasia. Advanced adenoma was defined as an adenoma of ≥ 10 mm or with a histological examination showing ≥ 25% villous component or high-grade dysplasia. If multiple lesions presented, the participant was classified by the most advanced one. When summarizing, advanced colorectal neoplasms (ACRN) were defined as a cancer or advanced adenoma, and overall colorectal neoplasms (CRN) were defined as a cancer or any adenoma.

### 2.3 Measurements and definitions

The HRFQ is a self-administered questionnaire, in which data on medical history and health-related behavior were filled by the participants while physical measurements and laboratory tests were performed by trained staffs. In the questionnaire, the smoking status was defined as “yes” (current/former) if they smoked at least one cigarette a day for more than 6 months, while the alcohol drinking status was defined as “yes” (current/former) if he/she had a drink at least once a week for more than 6 months. Additionally, a history of night work was defined as working three or more hours between 12 pm and 5 am at least once a month which lasted for more than half a year during their entire career, and sedentary work was referred as working in a sitting, reclining or lying posture in office, driving, or consoles etc. for more than half of the working time during their whole career. Moreover, history of diabetes was self-reported.

Some indicators for analysis were transformed from the collected data. Among them, age was calculated as (date of informed consent - date of birth)/365.25. The marriage status was collected according to four categories (married, unmarried, divorced, or windowed), but summarized into two (married or others). The education background was retrieved from five categories (illiteracy, primary school, secondary or middle school, college or undergraduate, or graduate student), but aggregated into three (primary school or below, secondary or middle school, or college or above). The Body mass index (BMI) was calculated by dividing measured weight (kg) by the square of the height (m^2^) and categorized according to the recommendation of Working Group on Obesity in China (25), where overweight or obesity was defined by BMI ≥ 24 kg/m^2^.

### 2.4 Data curation and statistical analysis

**Figure 1:**
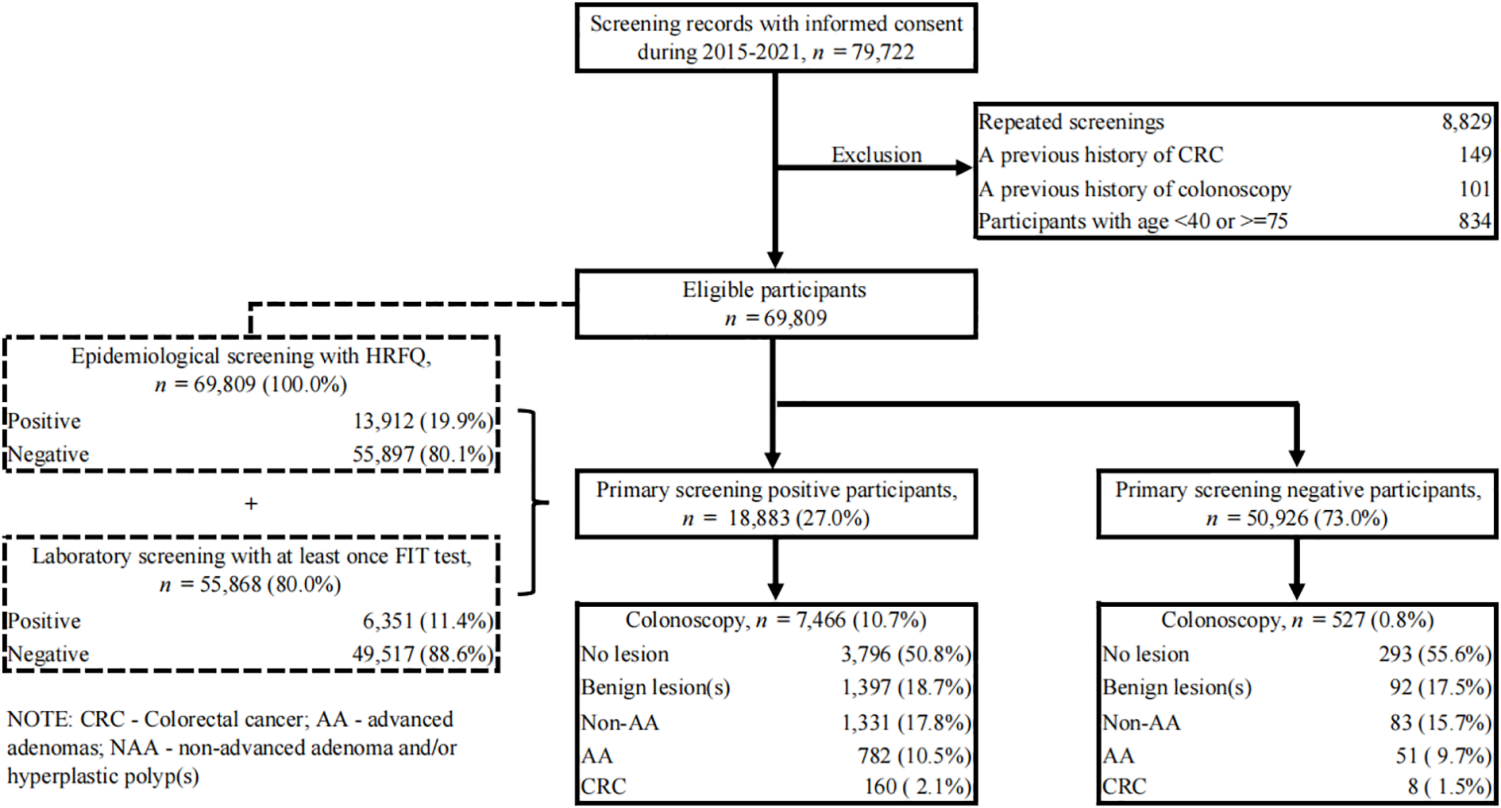
Flow diagram illustrating the selection of study subjects. CRC, colorectal cancer; AA, advanced adenomas; non-AA, non-advanced adenoma and/or hyperplastic polyp(s); HRFQ, high-risk factor questionnaire; FIT, fecal immunochemical test.

A total of 79,722 records during our study period were retrieved from the CRC screening system. Quality control was first performed on these data. Repeated screenings (n=8,829) were identified in advance by comparing their names and identification numbers, and only their earliest screening records were included, which ensures every single participant has their earliest CRC screening in their residential areas. Next, the following exclusion criteria were applied: a previous history of CRC (n=149), a previous history to undergo a colonoscopy, sigmoidoscopy, or barium contrast enema (n=101), and subjects with age<40 or >=75 (n=834). Finally, 69,809 participants were eligible and involved in our downstream analysis (**Figure 1**).

In the analysis, continuous variables were presented as mean ± SD (standard deviation), while categorical variables were described as the frequency with percentage. Our analysis focused on a special sub-population who underwent colonoscopy with negative primary screening results and the discovery of potential reasons for false negatives. To detect differences between groups, a t-test for continuous variables, and a Chi-square test or Fisher’s exact test for categorical variables were used. Additionally, to explore the reasons for colonoscopy uptake within this special sub-population, a retrospective telephone survey was conducted with an initial cohort of 199 participants from three communities, in late 2024.

Binary logistic regression models were conducted to compare the predictive ability of different screening methods. To assess robustness, we conducted sensitivity analyses. First, we repeated the analyses after excluding HRFQ-identified symptomatic individuals among screening-negative participants who underwent colonoscopy (e.g., chronic diarrhea, constipation, mucous/bloody stools, appendicitis or appendectomy, and cholecystitis or cholecystectomy). Second, to account for key confounders that might affect the performance of FIT and HRFQ, multivariable logistic regression model adjusting for age, sex, BMI, education, and marital status was applied. All analyses were performed in R with a version of 4.2.2 (26). A two-sided *P* value<0.05 was considered statistically significant. When multiple comparison occurred, a Bonferroni correction was applied.

## 3 Results

### 3.1 A comparable prevalence of CRN, ACRN, and CRC between primary screening negative and positive participants who underwent colonoscopy

Between 2015 and 2021, a total of 69,809 eligible residents aged 40 to 74 in Yuexiu District of Guangzhou (**Supplementary Figure S1**) attended the CRC screening. Only their first screening records were included in this study. All of them completed the HRFQ questionnaire with a positive rate of 19.9% and 80.0% of them underwent at least once FIT test with a positive rate of 11.4% (**Figure 1**). A summary of these results showed 18,883 (27.0%) participants were identified as primary screening positives, defined by positive HRFQ or at least one positive FIT result, and the other 50,926 (73.0%) were categorized as negatives. Of these participants, 7,466 with positive primary screening and 527 with negative primary screening underwent colonoscopy. Surprisingly, a comparable prevalence of benign lesion(s) (18.7% vs. 17.5%), non-advanced adenoma (17.8% vs. 15.7%), advanced adenoma (10.5% vs. 9.7%) and CRC (2.1% vs. 1.5%) was found between these two sub-populations, with a *P* value of 0.287 showing no between-group difference (**Table 1**).

**Table 1:** Comparison of colonoscopy results between primary screening negative and positive participants who underwent colonoscopy.

Summary	Total (n=7,993)	Primary screening		*P* value
		Negative (n=527)	Positive (n=7,466)	
Abnormal findings, n (%)				0.287^*^
No lesion	4,089 (51.2)	293 (55.6)	3,796 (50.8)	
Benign lesion(s)	1,489 (18.6)	92 (17.5)	1,397 (18.7)	
Non-AA	1,414 (17.7)	83 (15.7)	1,331 (17.8)	
AA	833 (10.4)	51 (9.7)	782 (10.5)	
CRC	168 (2.1)	8 (1.5)	160 (2.1)	
Overall colorectal neoplasms (CRN), n (%)				0.101^*^
Yes	2,415 (30.2)	142 (26.9)	2,273 (30.4)	
No	5,578 (69.8)	385 (73.1)	5,193 (69.6)	
Advanced colorectal neoplasms (ACRN), n (%)				0.376^*^
Yes	1,001 (12.5)	59 (11.2)	942 (12.6)	
No	6,992 (87.5)	468 (88.8)	6,524 (87.4)	
Colorectal cancer (CRC), n (%)				0.418^*^
Yes	168 (2.1)	8 (1.5)	160 (2.1)	
No	7,825 (97.9)	519 (98.5)	7,306 (97.9)	

^*^Chi-square test.

Benign lesions include intestinal polyp, enterelcosis, and non-adenomatous lesions.

AA, advanced adenomas; non-AA, non-advanced adenoma.

To investigate the differences in the prevalence of dysplasia at various levels between primary screening positive and negative participants who underwent colonoscopy, additional statistical comparisons were made (**Table 1**). Likewise, similar prevalence was found for CRN (30.4% vs. 26.9%, *P* = 0.101), ACRN (12.6% vs. 11.2%, *P* = 0.376), and CRC (2.1% vs. 1.5%, *P* = 0.418). These patterns persisted in a sensitivity analysis excluding HRFQ-identified symptomatic individuals from the screening-negative group, which yielded similar prevalence estimates (**Supplementary Table S1**). This motivated us to further explore the demographic and clinical characteristics of these primary-screening-negative participants to identify and distinguish them from other asymptomatic populations for improvement in CRC screening programs.

### 3.2 Basic characteristics of participants who had negative primary screening but underwent colonoscopy, in contrast with those who had positive primary screening

Basic characteristics of subjects who underwent colonoscopy were first elucidated and compared between participants with negative and positive primary screening results (**Table 2**). Of the 7,993 participants who did colonoscopy, 41.7% were men and their mean age was 59.9 ± 8.1 years. The comparison analysis demonstrated that among the population with colonoscopy for CRC diagnosis, the proportions of participants who were current or past smokers (4.7% vs. 10.4%, *P* < 0.001), had a history of night work (13.3% vs. 23.2%, *P* < 0.001), and had a sedentary job (26.9% vs. 44.2%, *P* < 0.001) were lower in subjects who got negative primary screening results than in those with positive primary screening results. Except for these, no significant between-group differences were found for other characteristics. The CRC risk assessment under the current screening protocol showed all the primary screening negative participants who did colonoscopy reached the low CRC risk criteria whereas 29.0% of them had one of the risk conditions (**Supplementary Table S2**). Similar results were found for comparison between primary screening negative participants who did colonoscopy and all with positive primary screening (**Supplementary Table S3**).

**Table 2:** Demographic comparison between primary screening negative and positive participants who underwent colonoscopy.

Summary	Total (n=7,993)	Primary screening		*P* value
		Negative (n=527)	Positive (n=7,466)	
Gender, n (%)				0.228^*^
Male	3,332 (41.7)	206 (39.1)	3,126 (41.9)	
Female	4,661 (58.3)	321 (60.9)	4,340 (58.1)	
Age (years), Mean ± SD				0.879^$^
	59.9 ± 8.1	59.8 ± 7.9	59.9 ± 8.1	
Age group (years), n (%)				0.632^*^
40-49	989 (12.4)	64 (12.1)	925 (12.4)	
50-59	2,512 (31.4)	178 (33.8)	2,334 (31.3)	
60-69	3,636 (45.5)	234 (44.4)	3,402 (45.6)	
70-74	856 (10.7)	51 (9.7)	805 (10.8)	
Marriage status, n (%)				0.821^*^
Married	7,309 (91.4)	480 (91.1)	6,829 (91.5)	
Other	684 (8.6)	47 (8.9)	637 (8.5)	
Education level, n (%)				0.597^*^
Primary school or below	641 (8.0)	43 (8.2)	598 (8.0)	
Secondary or middle school	5,245 (65.6)	355 (67.4)	4,890 (65.5)	
College or above	2,107 (26.4)	129 (24.5)	1,978 (26.5)	
BMI (kg/m^2^), Mean ± SD				0.853^$^
	23.49 ± 3.31	23.46 ± 3.19	23.49 ± 3.31	
Overweight or obesity, n (%)				1.000^*^
	3,296 (41.2)	217 (41.2)	3,079 (41.2)	
Current/ex-smoker, n (%)				<0.001^*^
	798 (10.0)	25 (4.7)	773 (10.4)	
Alcohol drinking, n (%)				0.828^*^
	217 (2.7)	13 (2.5)	204 (2.7)	
A history of night work, n (%)				<0.001^*^
	1,805 (22.6)	70 (13.3)	1,735 (23.2)	
Sedentary more than half in work time, n (%)				<0.001^*^
	3,440 (43.0)	142 (26.9)	3,298 (44.2)	
History of diabetes, n (%)				0.085^*^
	753 (9.4)	38 (7.2)	715 (9.6)	

^*^Chi-square test; ^$^Independent *t* test.

### 3.3 Characteristics comparison between primary screening negative participants who did or did not undergo colonoscopy

To investigate why participants who were negative in primary screening might benefit from a colonoscopy, baseline characteristics of subjects who did and did not undergo colonoscopy were compared (**Table 3**). The results demonstrated that among participants with primary screening negative results, the proportion of middle-aged adults less than 60 (45.9% vs. 37.3%, *P* < 0.001) and people with college or above education background (24.5% vs. 17.2%, *P* < 0.001) was higher in subjects who did colonoscopy than those who did not. Moreover, participants that opted for a colonoscopy showed higher rates for choosing healthy behaviors, including less smoking (4.7% vs. 8.5%, *P=*0.002), having a history of night work (13.3% vs. 18.1%, *P* = 0.005), having sedentary work (26.9% vs. 35.4%, *P* < 0.001), and having a lower rate of diabetes (7.2% vs. 11.5%, *P* = 0.003); but more of them had one risk factor for CRC (29.0% vs. 22.1%, *P<*0.001), with significant differences in a history of chronic diarrhea (5.1% vs. 2.0%, *P* < 0.001), chronic constipation (9.3% vs. 4.7%, *P* < 0.001), or mucoid or bloody feces (5.7% vs. 3.7%, *P* = 0.020) in the past two year. To further explore the reasons for colonoscopy uptake among participants with negative primary screening, a retrospective telephone survey was conducted, but yielded a low response rate (13.1%, 26/199). Over half of the respondents (53.8%) could not recollect their reasons for pursuing the colonoscopy, while 26.9% reported self-perceived abnormalities in bowel movements (**Supplementary Table S4**).

**Table 3:** Demographic comparison of primary screening negative participants with or without colonoscopy.

Summary	Total (n=50,926)	PS negative participants		*P* value
		With colonoscopy (n=527)	Without colonoscopy (n=50,399)	
Gender, n (%)				0.522^*^
Male	19,174 (37.7)	206 (39.1)	18,968 (37.6)	
Female	31,752 (62.3)	321 (60.9)	31,431 (62.4)	
Age (years), Mean ± SD				<0.001^$^
	61.2 ± 8.1	59.8 ± 7.9	61.2 ± 8.1	
Age group (years), n (%)				<0.001^*^
40-49	5,260 (10.3)	64 (12.1)	5,196 (10.3)	
50-59	13,797 (27.1)	178 (33.8)	13,619 (27.0)	
60-69	24,170 (47.5)	234 (44.4)	23,936 (47.5)	
70-74	7,699 (15.1)	51 (9.7)	7,648 (15.2)	
Marriage status, n (%)				0.298^*^
Married	47,042 (92.4)	480 (91.1)	46,562 (92.4)	
Other	3,884 (7.6)	47 (8.9)	3,837 (7.6)	
Education level, n (%)				<0.001^*^
Primary school or below	6,747 (13.2)	43 (8.2)	6,704 (13.3)	
Secondary or middle school	35,405 (69.5)	355 (67.4)	35,050 (69.5)	
College or above	8,774 (17.2)	129 (24.5)	8,645 (17.2)	
BMI (kg/m^2^), Mean ± SD				0.358^*^
	23.62 ± 3.33	23.46 ± 3.19	23.62 ± 3.33	
Overweight or obesity, n (%)				0.672^*^
	21,383 (42.0)	216 (41.0)	21,167 (42.0)	
Current/ex-smoker, n (%)				0.002^*^
	4,327 (8.5)	25 (4.7)	4,302 (8.5)	
Alcohol drinking, n (%)				0.557^*^
	1,026 (2.0)	13 (2.5)	1,013 (2.0)	
A history of night work, n (%)				0.005^*^
	9,192 (18.0)	70 (13.3)	9,122 (18.1)	
Sedentary more than half in work time, n (%)				<0.001^*^
	17,983 (35.3)	142 (26.9)	17,841 (35.4)	
History of diabetes, n (%)				0.003^*^
	5,834 (11.5)	38 (7.2)	5,796 (11.5)	
Risk factors				
Chronic diarrhea, n (%)				<0.001^*^
	1,040 (2.0)	27 (5.1)	1,013 (2.0)	
Chronic constipation, n (%)				<0.001^*^
	2,425 (4.8)	49 (9.3)	2,376 (4.7)	
Mucoid or bloody feces, n (%)				0.020^*^
	1,881 (3.7)	30 (5.7)	1,851 (3.7)	
Chronic appendicitis or appendectomy, n (%)				0.109^*^
	1,989 (3.9)	13 (2.5)	1,976 (3.9)	
Chronic cholecystitis or cholecystectomy, n (%)				1.000^*^
	932 (1.8)	10 (1.9)	922 (1.8)	
Traumatic experience in the past 20 years, n (%)				0.211^*^
	3,020 (5.9)	24 (4.6)	2,996 (5.9)	
Number of risk factors, n (%)				<0.001^*^
0	39,639 (77.8)	374 (71.0)	39,265 (77.9)	
1	11,287 (22.2)	153 (29.0)	11,134 (22.1)	

^*^Chi-square test; ^$^Independent *t* test.

PS, primary screening.

### 3.4 Comparison of detection rates for CRN, ACRN, and CRC among different screening methods

**Figure 2:**
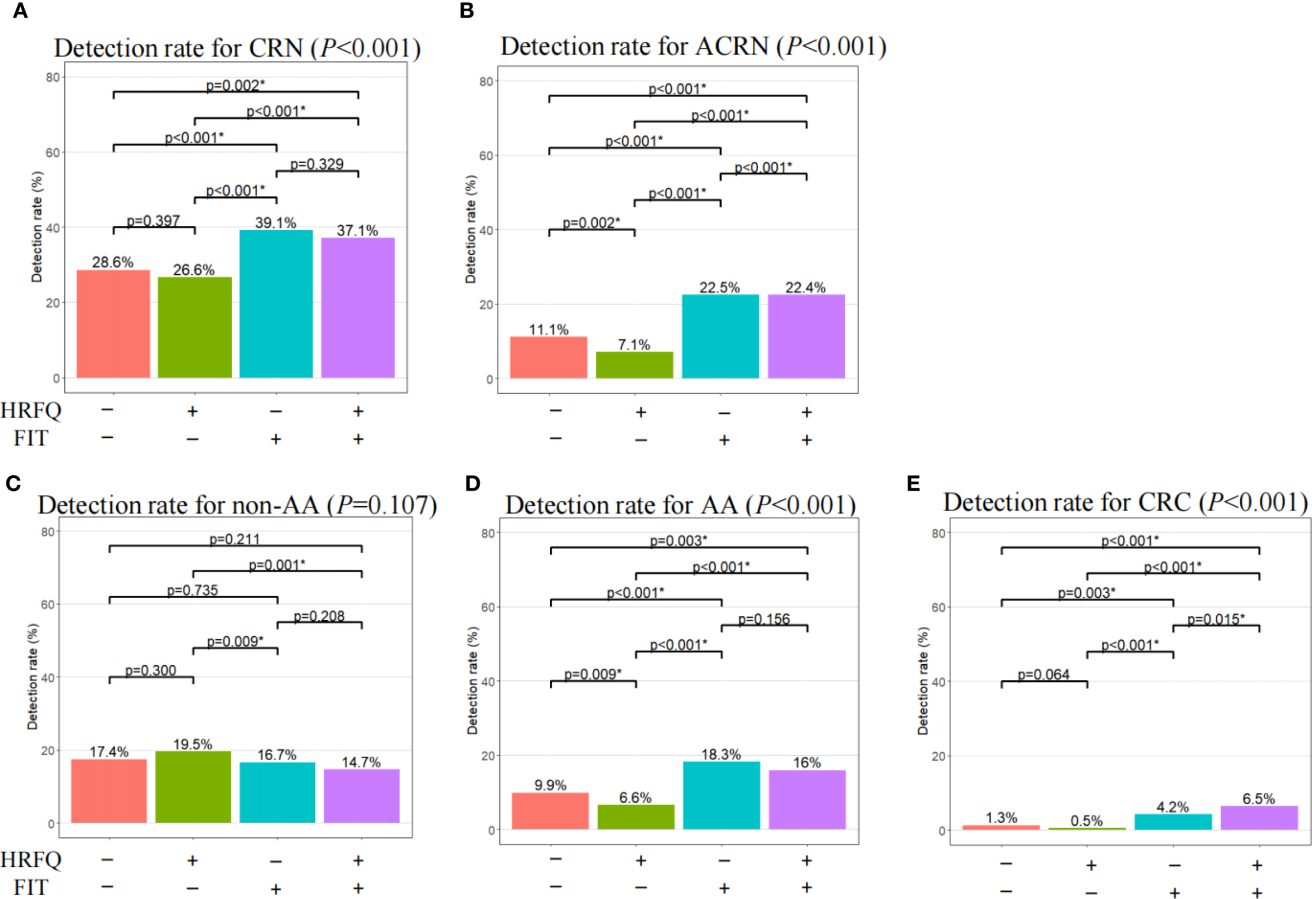
Comparison of detection rate for CRN, ACRN, non-AA, AA, and CRC among different screening methods. CRN, overall colorectal neoplasms; ACRN, advanced colorectal neoplasms; CRC, colorectal cancer; AA, advanced adenomas; non-AA, non-advanced adenoma and/or hyperplastic polyp(s); HRFQ, high-risk factor questionnaire; FIT, Fecal immunochemical test. ^*^Statistically significant after a Bonferroni correction of the original test criterion of 0.05.

Further, we evaluated the detection ability of different screening methods for all grades of colorectal dysplasia to discover their contribution in lesion identification. Among participants undergoing colonoscopy, the overall detection rates for CRN, ACRN and CRC under current screening protocol were 30.2%, 12.5% and 2.1%, respectively (**Supplementary Table S5**). Relative to negative FIT, positive FIT displayed higher detection rates for CRN and ACRN regardless of HRFQ results (**Figures 2A, B**), which were largely attributed to enhanced detection rates for AA and CRC but not non-AA (**Figures 2C–E**). This suggested FIT positive had a better risk predictive ability for AA and CRC. However, positive HRFQ showed an opposite trend. That is, negative HRFQ showed higher or close detection rates for CRN and ACRN whenever FIT was negative or not (**Figures 2A, B**). This might be mainly ascribed to the declining predictive ability of HRFQ for non-AA and AA but not CRC (**Figure 2C–E**). All these suggested that positive HRFQ does not have discriminatory ability for CRC, as expected.

**Figure 3:**
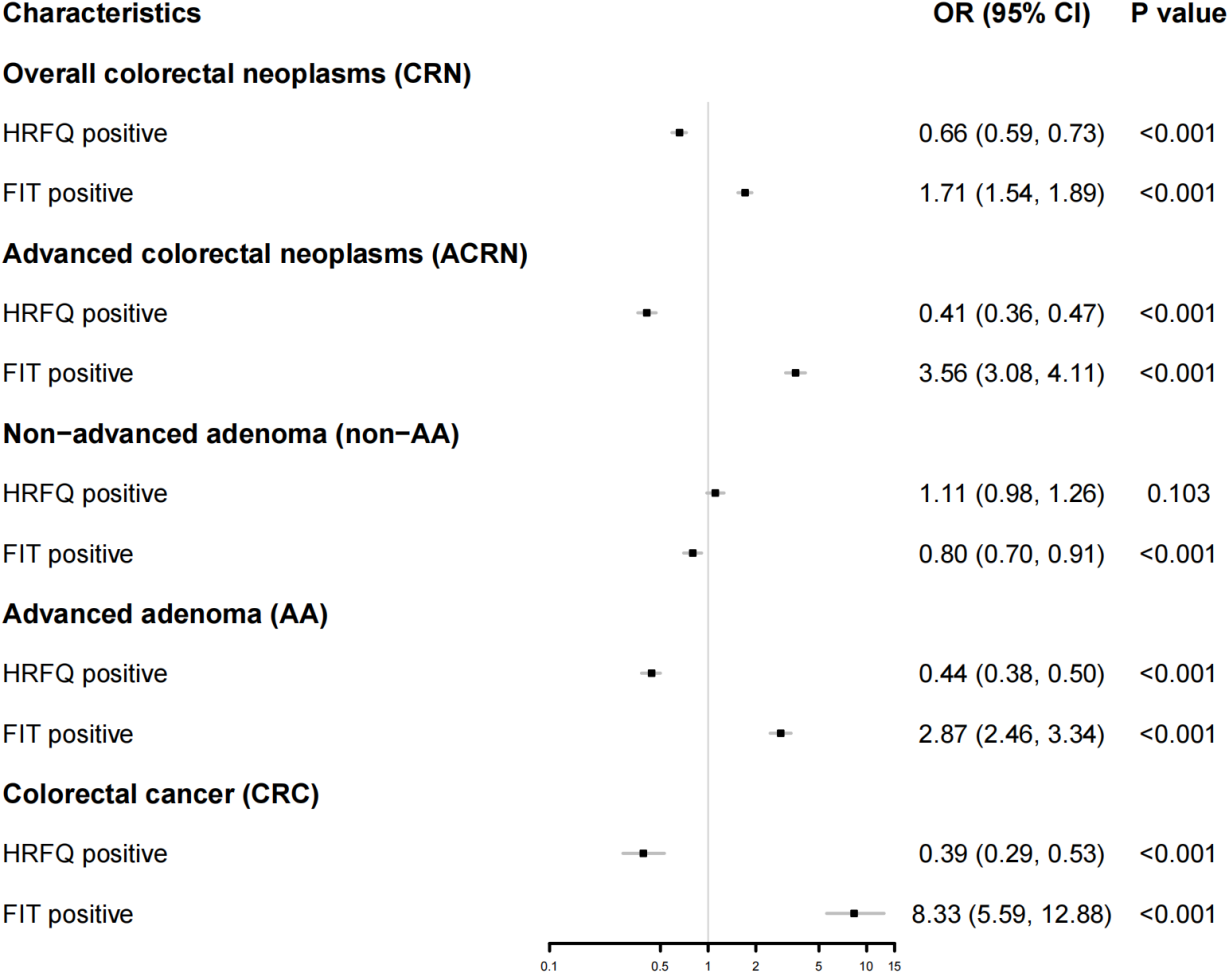
Forest map showing the result from a logistic regression for the predictive ability of FIT or HRFQ on CRN, ACRN, non-AA, AA, and CRC. CRN, Overall colorectal neoplasms; ACN, Advanced colorectal neoplasms; CRC, Colorectal cancer; AA, advanced adenomas; NAA, non-advanced adenoma and/or hyperplastic polyp(s); HRFQ, high-risk factor questionnaire; FIT, Fecal immunochemical test.

The logistic regression demonstrated consistent results (**Figure 3**). That is, FIT positives had significantly higher predictive ability for AA (OR: 2.87; 95% CI: [2.46, 3.34]) and CRC (OR: 8.33; 95% CI: [5.59, 12.88]), CRN (OR: 1.71; 95% CI: [1.54, 1.89]) and ACRN (OR: 3.56; 95% CI: [3.08 4.11]). Nevertheless, HRFQ positives showed significantly lower predictive ability for AA (OR: 0.44; 95% CI: [0.38, 0.50]) and CRC (OR: 0.39; 95% CI: 0.29, 0.53]), and CRN (OR: 0.66; 95% CI: [0.59, 0.73]), and ACRN (OR: 0.41; 95% CI: [0.36, 0.47]). Multivariable logistic models adjusting for age, sex, BMI, education, and marital status showed similar estimates, reinforcing the robustness of these findings (**Supplementary Figure S2**).

## 4 Discussion

The nationwide CRC screening protocol that combines a risk stratification system of HRFQ and double FITs as the first stage of screening and colonoscopy as the second stage of screening has been applied for several years since 2006 in China, but its effectiveness and conveniences are controversial (27). Our retrospective study, conducted in a high-developed city in southern China, focused on a sub-group of participants who had both negatives of HRFQ and FITs but showed a comparable prevalence of colorectal lesions with HRFQ or FIT positive subjects (**Figure 1**, **Table 1**). After comparison analysis with high-risk participants defined by current screening strategy, we found that the risk predictive ability for AA (*P* < 0.001), CRC (*P* < 0.001), CRN (*P* < 0.001) and ACRN (*P* < 0.001) in HRFQ positives was similar or even less effective than those in HRFQ negatives regardless of FIT results (**Figure 3**), which suggested a potential inversion of risk stratification for colorectal neoplasia. Such a misclassification might give subjects a false confidence in the diagnostics and lead to an elevated risk of missed diagnoses. To our knowledge, our study was the first to systematically evaluate the prevalence of colorectal lesions in this specific subgroup, and to highlight the missed diagnosis problem in the current CRC risk screening protocol in China.

Further analyses comparing the basic characteristics and CRC risk factors of participants revealed that the sub-population of interest possessed less risk factors of CRC, which means a lower CRC risk than those who had HRFQ or FIT positives (**Table 2**). In comparison to participants with negative screens who opted out of colonoscopy, individuals that underwent the colonoscopy with negative screens displayed significantly younger age, higher educational achievements, and a preference for healthier behaviors. However, they exhibited a greater likelihood of having one risk factor for CRC (**Table 3**). These results indicated that the younger people with better education level might have higher health beliefs and self-perception of the risks for CRC. Similar analyses uncovered strong associations between a behavior change and the choice for colonoscopy (13). Meanwhile, although a retrospective telephone survey was conducted to further explore the reasons for colonoscopy in this sub-population, the study was limited by the low response rate (13%) and significant recall bias, which were intrinsic to retrospective study designs. A prospective study to compare the colonoscopy result between primary screening positive and negative should be considered in the future.

The current CRC screening protocol in China has caused many of the high-risk participants to be excluded in the first screening stage (15). FIT has been shown to be effective in high-risk predictiveness for CRC and AA in asymptomatic populations (9), and this work supports our finding (**Figures 2**, **3**). The HRFQ serves to enrol more high-risk participants who may have false-negative FIT results, and thus detect more intestinal diseases (14). However, our work showed that current risk stratification strategy using HRFQ did not provide higher detection rates for AA, CRC, and thus ACRN in contrast with a group of active screening participants who have both negatives of HRFQ and FIT. This suggested that the predictive ability of HRFQ for high-risk colorectal lesions in FIT false-negative population was insufficient, which may attribute to the indirect or heterogeneous links between the included items in HRFQ [e.g., appendicitis/appendectomy (28), cholelithiasis/cholecystitis or cholecystectomy (29), and traumatic life events (30)] with CRC. Prior studies showed that removing these subitems changed detection little, whereas a history of colorectal adenoma contributed most of the predictive yield (27). Another reason was that HRFQ does not explicitly include some important CRC risk factor (e.g., age, BMI, smoking, alcohol intake, and dietary pattern), which constrained performance. Although combining HRFQ with FIT improves yield, HRFQ alone remains limited (31). In light of these findings, HRFQ could be strengthened by adding established CRC determinants (e.g., age, BMI, smoking, alcohol intake, dietary pattern rather than relying on items with indirect links). This direction aligns with previous work that most of risk stratification system tried to designed a new scoring system based on the traditional CRC risk factors like smoking, age, sex, using a retrospective re-assessment of collected data (14, 20, 27, 32). On the other hand, some studies tried to combine some newly identified risk factors (e.g., body mass index) to improve the traditional scoring system (e.g., the Asia-Pacific Colorectal Screening score) for risk stratification of colorectal lesions (33, 34), or develop a new risk prediction score based on the updated knowledge of CRC. For example, based on the China Kadoorie Biobank (CKB), Hang et al. developed a novel risk prediction tool specifically tailored for Chinese population to identify individuals at high risk for CRC (17), considering the age, sex, education level, smoking status and pack-years, alcohol drinking, dietary factors, physical activity, BMI, prevalent diabetes, history of peptic ulcer disease, gallstone disease, and family history of cancer in first-degree relatives. The finding that the use of newly identified risk factors can improve the predictive ability of CRC, highlights the request for updating the current screening strategy. In parallel, the weights of HRFQ components should be recalibrated with multivariable/penalized models so that higher-yield items contribute more to the overall score. Additionally, efforts also have been made to improve the effectiveness of FIT. Some studies have discussed to lower the cut-off value for FIT to increase sensitivity (35) and adopt a tailed FIT threshold strategy (10), however, these strategies may reduce specificity and increase colonoscopy burden, or enhance difficulty in practices.

Some limitations were also acknowledged. First, the sub-population that tested negative on both the HRFQ and FIT yet underwent colonoscopy was relatively small in this work (~1%) and highly selective (e.g., symptom-driven self-selection), which might limit the representative of the broader screening-negative population. Despite we may overestimate the prevalence of colorectal lesions in this subgroup, a similar high risk of colorectal neoplasms was also observed in over 5 million screening participants in such a sub-population (15). Thus, extended research about these people in other regions is highly recommended. Second, the detailed reasons why these participants with negative primary screening volunteered for colonoscopy remain unclear. We were unable to obtain some CRC risk features like a history of medications and laboratory measurements (18) due to the retrospective nature, which may compromise the predictive capability of the HRFQ. Third, this study was conducted within an urban screening programme with comparatively greater access to endoscopy and health services; therefore, estimates of uptake and lesion prevalence may not generalize to rural or resource-limited settings. External validation across diverse regions and healthcare tiers in China is warranted. Finally, the unavailability of colonoscopy quality indicators such as adenoma detection rate (ADR) compromised the reliability of the colonoscopy results, while the lack of some lesion features like location and size limited further investigation into which types of lesions are most likely to be missed by FIT or HRFQ. However, findings in this work did remind us of the severe problem of a missed diagnosis, suggesting that an improvement to CRC screening strategy is urgently needed.

## 5 Conclusion

Despite a potential overestimation of the detection rate of colorectal lesions among the primary screening-negative population, these special populations with active screening willingness to undergo a colonoscopy should be considered for earlier colonoscopy. At the primary-care level, for HRFQ/FIT-negative patients with persistent or progressive gastrointestinal symptoms or strong screening intention after counselling, clinicians should consider short-interval re-assessment (including repeat FIT where available) and maintain a low threshold for diagnostic colonoscopy, adapted to local endoscopy capacity. In this subgroup, the primary screening negatives might be attributed to the HRFQ’s insufficient predictive ability for the risk of colorectal lesions. Our findings highlight the urgent need to reassess and improve the CRC risk scoring system and to contribute to the enhancement of CRC screening strategies.

## Data Availability

The data analyzed in this study is subject to the following licenses/restrictions: The datasets presented in this article are not readily available because the CDC does not encourage to share the original data. Requests to access these datasets should be directed to YL, liuy683@gzucm.edu.cn.
