# Supplementary material for: Colonoscopy outcomes of primary screening negative participants highlight the missed diagnosis problem of colorectal cancer screening: an observational study from Yuexiu district in Guangzhou, China
Source: Front Oncol. 2025 Oct 21;15:1642326. doi: 10.3389/fonc.2025.1642326 (PMC12582931; doi:10.3389/fonc.2025.1642326)
Supplement: Supplementary file 1 [file DataSheet1.pdf]

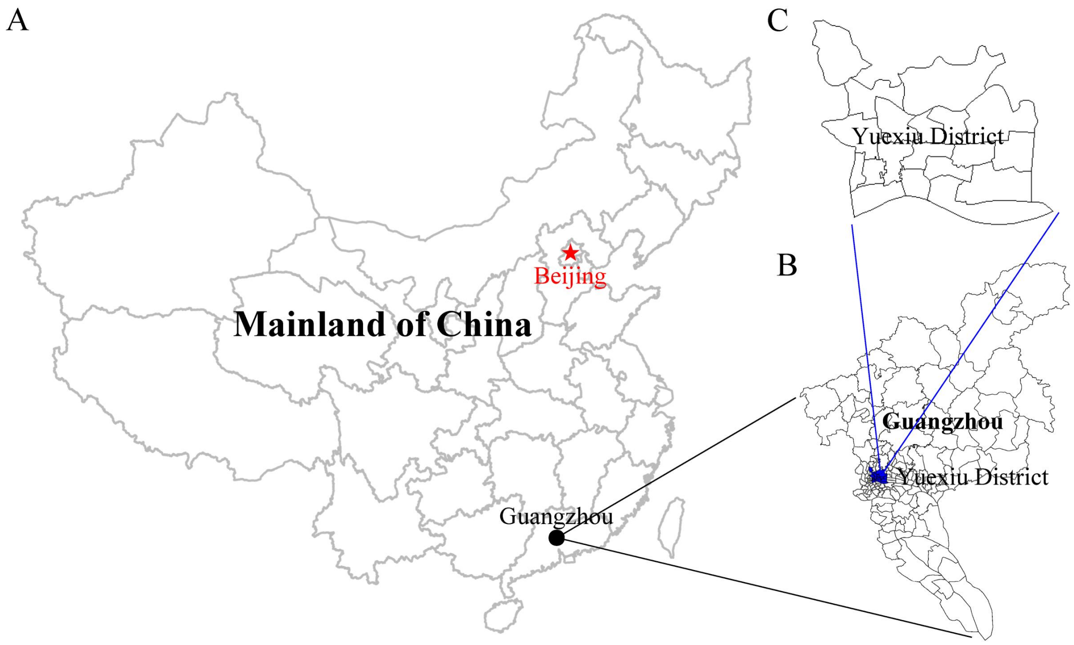

FIGURE S1. Location of the study area in China. Our study area located in the south of Chinese mainland (A). More precisely, they located in Yuexiu District of Guangzhou (B), a city known as the heart of the Guangdong-Hong Kong-Macau Greater Bay Area. Data from three communities of Dadong, Dengfeng and Liurong (C) were collected.

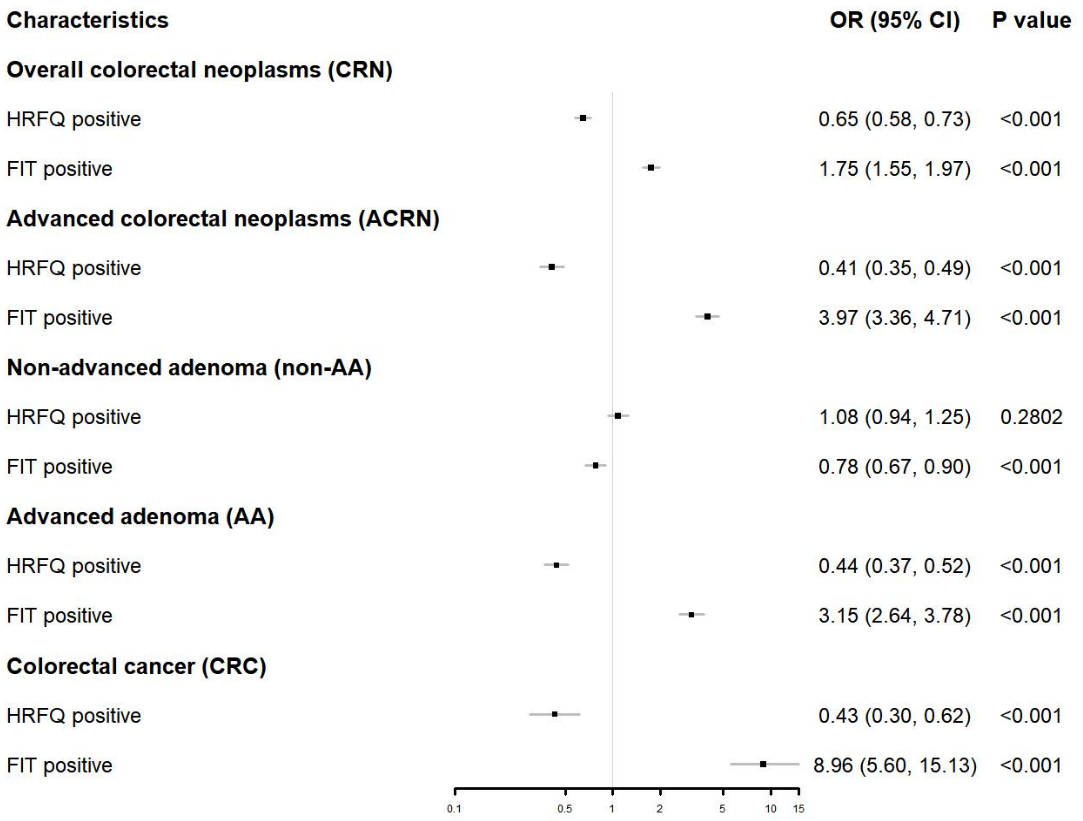

FIGURE S2. Forest map showing the result from a logistic regression for the predictive ability of FIT or HRFQ on CRN, ACRN, non-AA, AA, and CRC adjusted for age, gender, BMI, education, and marital status.

CRN - Overall colorectal neoplasms; ACN - Advanced colorectal neoplasms; CRC - Colorectal cancer; AA - advanced adenomas; NAA - non-advanced adenoma and/or hyperplastic polyp(s); HRFQ - high-risk factor questionnaire; FIT - Fecal immunochemical test.

TABLE S1. Comparison of colonoscopy results between primary screening positive and negative participants without symptoms who underwent colonoscopy

Summary		Total	Primary screening		*P* value
			Negative	Positive	
Total number		7,993	398	7,446	
Abnormal findings, n (%)					0.771[&]
	No lesion	4,089 (51.1)	213 (53.5)	3,796 (50.8)	
	Benign lesion(s)	1,489 (18.6)	72 (18.1)	1,397 (18.7)	
	Non-AA	1,414 (17.6)	65 (16.3)	1,331 (17.8)	
	AA	833 (10.4)	42 (10.6)	782 (10.5)	
	CRC	168 (2.1)	6 (1.5)	160 (2.1)	
Overall colorectal neoplasms (CRN), n (%)					0.417[&]
	Yes	2,415 (30.2)	113 (28.4)	2,273 (30.4)	
	No	5,578 (69.8)	285 (71.6)	5,193 (69.6)	
Advanced colorectal neoplasms (ACRN), n (%)					0.804[&]
	Yes	1,001 (12.5)	48 (12.1)	942 (12.6)	
	No	6,992 (87.5)	350 (87.9	6,524 (87.4)	
Colorectal cancer (CRC), n (%)					0.496[&]
	Yes	168 (2.1)	6 (1.5)	160 (2.1)	
	No	7,825 (97.9)	392 (98.5)	7,306 (97.9)	

&: Chi-square test.

Benign lesions include intestinal polyp, enterelcosis, and non-adenomatous lesions

CRC - Colorectal cancer; AA - advanced adenomas; non-AA - non-advanced adenoma and/or hyperplastic polyp(s)

TABLE S2. Comparison of risk factors for CRC between primarity screening negative and positive paticipants who underwent colonoscopy

Characteristics	Participants with colonoscopy		*P* value
	Primary screening negative	Primary screening positive	
Total number	527	7,466	
A history of cancer, n (%)			<0.001&
	0 (0.0)	344 (4.6)	
A history of polys, n (%)			<0.001&
	0 (0.0)	1,754 (23.5)	
A family history of CRC in a first-degree relative, n (%)			<0.001&
	0 (0.0)	1,502 (20.1)	
Risky conditions			
Chronic diarrhea, n (%)			<0.001*
	27 (5.1)	1,429 (19.1)	
Chronic constipation, n (%)			<0.001*
	49 (9.3)	1,828 (24.5)	
Mucoid bloody feces, n (%)			<0.001*
	30 (5.7)	2,395 (32.1)	
Chronic appendicitis or appendectomy, n (%)			<0.001*
	13 (2.5)	675 (9.0)	
Chronic cholecystitis or cholecystectomy, n (%)			<0.001*
	10 (1.9)	530 (7.1)	
Traumatic experience in the past 20 years, n (%)			<0.001*
	24 (4.6)	1,693 (22.7)	
Number of risky conditions, n (%)			<0.001&
0	374 (71.0)	2,683 (35.9)	
1	153 (29.0)	1,757 (23.5)	
>=2	0 (0.0)	3,026 (40.5)	
At least one positive FIT result, n (%)			<0.001&
	0 (0.0)	2,779 (41.3)	

*: Chi-square test was used; &: Fisher's exact test was used; $: Independent t test was used.

TABLE S3. Demographic comparison between primarity screening negative participants with colonoscopy and all primarity screening positive participants

Characteristics	Primary screening negative participants with colonoscopy	All primary screening positive participants	*P* value
Total number	527	18,883	
Gender, n (%)			0.855*
Male	206 (39.1)	7,474 (39.6)	
Female	321 (60.9)	11,409 (60.4)	
Age (years), Mean ± SD			<0.001$
	59.8 ± 7.9	61.1 ± 8.1	
Age group (years), n (%)			<0.001*
40-49	64 (12.1)	2,024 (10.7)	
50-59	178 (33.8)	5,083 (26.9)	
60-69	234 (44.4)	9,051 (47.9)	
70-74	51 (9.7)	2,725 (14.4)	
Marriage status, n (%)			0.484*
Married	480 (91.1)	17,006 (90.1)	
Other	47 (8.9)	1,877 (9.9)	
Education level, n (%)			0.358*
Primary school or below	43 (8.2)	1,890 (10.0)	
Secondary or middle school	355 (67.4)	12,356 (65.4)	
College or above	129 (24.5)	4,637 (24.6)	
BMI (kg/m2), Mean ± SD			0.551$
	23.46 ± 3.19	23.56 ± 3.40	
Overweight or obesity, n (%)			0.813*
	216 (41.0)	7,855 (41.6)	
Current/ex-smoker, n (%)			<0.001*
	25 (4.7)	2,026 (10.7)	
Alcohol drinking, n (%)			0.889*
	13 (2.5)	503 (2.7)	
A history of night work, n (%)			<0.001*
	70 (13.3)	4,400 (23.3)	
Sedentary more than half in work time , n (%)			<0.001*
	142 (26.9)	8,195 (43.4)	
History of diabetes, n (%)			0.003*
	38 (7.2)	2,153 (11.4)	
A history of cancer, n (%)			<0.001&
	0 (0.0)	1,318 (7.0)	
A history of polys, n (%)			<0.001&
	0 (0.0)	4,151 (22.0)	
A family history of CRC in a first-degree relative, n (%)			<0.001&

	0 (0.0)	3,641 (19.3)	
Risky conditions			
Chronic diarrhea, n (%)			<0.001*
	27 (5.1)	2,905 (15.4)	
Chronic constipation, n (%)			<0.001*
	49 (9.3)	4,265 (22.6)	
Mucoid bloody feces, n (%)			<0.001*
	30 (5.7)	5,159 (27.3)	
Chronic appendicitis or appendectomy, n (%)			<0.001*
	13 (2.5)	2,079 (11.0)	
Chronic cholecystitis or cholecystectomy, n (%)			<0.001*
	10 (1.9)	1,515 (8.0)	
Traumatic experience in the past 20 years, n (%)			<0.001*
	24 (4.6)	4,308 (22.8)	
Number of risky conditions, n (%)			<0.001&
0	374 (71.0)	7,529 (39.9)	
1	153 (29.0)	4,188 (22.2)	
>=2	0 (0.0)	7,166 (37.9)	

*: Chi-square test ; &: Fisher's exact test; $: Independent t test.

TABLE S4. The reason for colonoscopy after a negative primary screening result, among 26 sub-populations

Subjects	Why did you choose to conduct the colonoscopy after a negative primary screening result
Participants 1	Out of concern for one's physical health
Participants 2	Forgot
Participants 3	Forgot
Participants 4	Forgot
Participants 5	Just for a free body check
Participants 6	Out of concern for one's physical health
Participants 7	Forgot
Participants 8	Felt that there was an abnormality in my bowel movements (e.g., shape or frequency)
Participants 9	Forgot
Participants 10	Just for a free body check
Participants 11	Felt Forgot
Participants 12	Forgot
Participants 13	Felt that there is an abnormality in my bowel movements (e.g., shape or frequency)
Participants 14	Forgot
Participants 15	Felt that there is an abnormality in my bowel movements (e.g., shape or frequency)
Participants 16	Felt that there is an abnormality in my bowel movements (e.g., shape or frequency)
Participants 17	Forgot
Participants 18	Forgot
Participants 19	Felt that there was an abnormality in my bowel movements (e.g., shape or frequency)
Participants 20	Forgot
Participants 21	Forgot
Participants 22	Felt that there was an abnormality in my bowel movements (e.g., shape or frequency)
Participants 23	Forgot
Participants 24	Out of concern for one's physical health
Participants 25	Felt that there was an abnormality in my bowel movements (e.g., shape or frequency)
Participants 26	Forgot

TABLE S5. Comparison of colonoscopy results among different screening methods

Screening methods	Colonoscopy	Overall colorectal neoplasms		Advanced colorectal neoplasms		Non-advanced adenoma		Advanced adenoma		CRC	
		n	%	n	%	n	%	n	%	n	%
Overall	7,993	2,415	30.2%	1,001	12.5%	1,414	17.7%	833	10.4%	168	2.1%
HRFQ(-) + No_FIT	83	17	20.5%	7	8.4%	10	12.0%	5	6.0%	2	2.4%
HRFQ(-) + FIT(-)	476	136	28.6%	53	11.1%	83	17.4%	47	9.9%	6	1.3%
HRFQ(-) + FIT(+)	1,927	754	39.1%	433	22.5%	321	16.7%	352	18.3%	81	4.2%
HRFQ(+) + No_FIT	735	148	20.1%	39	5.3%	109	14.8%	36	4.9%	3	0.4%
HRFQ(+) + FIT(-)	3,920	1,044	26.6%	278	7.1%	766	19.5%	257	6.6%	21	0.5%
HRFQ(+) + FIT(+)	852	316	37.1%	191	22.4%	125	14.7%	136	16.0%	55	6.5%
